# COVID and depression among stratified population groups: a narrative review

**DOI:** 10.1186/s40001-023-01213-4

**Published:** 2023-08-03

**Authors:** Charul, Jyotdeep K. Raina, Sourabh Sharma, Shash Pal, Vikas Dogra, Parvinder Kumar, Rakesh K. Panjaliya

**Affiliations:** 1 Department of Zoology, University of Jammu, Jammu and Kashmir, 180006 India; 2 Department of Zoology, Govt. College for Women, Parade Ground, Jammu, Jammu and Kashmir 180001 India; 3 GGM Science College, Cluster University of Jammu, Jammu and Kashmir, 180001 India; 4 Institute of Human Genetics, University of Jammu, Jammu and Kashmir, 180006 India

**Keywords:** Covid-19, Depression, Mental health

## Abstract

The Covid-19 pandemic has impacted and infiltrated every aspect of our lives. Successive lockdowns, social distancing measures, and reduction in economic activity have developed a new way of living and, in many cases, tend to lead to depression. The initial strict lockdown for about 3 months and eventually for a few more months has imposed greater challenges on children and adolescents in terms of psychological problems and psychiatric disorders. Regardless of their viral infection status, many people have been affected by the psychosocial changes associated with the Covid-19 pandemic. In the present review, we have attempted to evaluate the impact of COVID on the mental health of people from different age groups and occupations. The present review has highlighted the need for taking effective measures by the stakeholder to cope with depression among human population groups worldwide.

## Introduction

An outburst of pneumonia caused by an unfamiliar virus was observed in Wuhan, China, in the later parts of 2019. The first appearance of this viral infection was reported on 17 November 2019 [1]. The etiological agent was at first named as 2019 novel coronavirus (2019-nCoV), but afterward, it was called Severe Acute Respiratory Syndrome Coronavirus 2 (SARS-CoV-2) [2]. The virus spread to almost all continents at a rapid pace, and it was in March 2020 that the World Health Organization (WHO) announced the spread as a pandemic and the disease was called Coronavirus Disease-2019 (COVID-19) [3]. Till 14th October 2021, the world has reported 239,905,767 coronavirus cases and 4,888,696 deaths [4]. The disease spreads via the droplet route and the infection ranges from asymptomatic to a proper disease marked by pneumonia, breathing difficulties, dry cough, diarrhea, fever, headache, and insomnia. The mortality rate ranges from 3–9% of all cases [1].

As per the International Committee on Taxonomy of Viruses (ICTV), SARS-CoV-2 belongs to the family Coronaviridae and order Nidovirales. Coronaviridae contains two subfamilies, i.e., Coronavirae and Torovirinae. Coronaviridae comprises four genera: (I) alpha coronavirus, NL63, and 229E; (II) beta coronavirus, SARS-CoV, SARS-CoV-2, HCoV-OC43, MERS-CoV; (iii) gamma coronavirus comprises viruses of birds and whales, and (iv) delta coronaviruses includes viruses of pigs and birds [5].

Coronaviruses were not known to infect humans till the outburst of SARS in 2002–2003 caused by SARS-CoV. A decade later, MERS was identified in Saudi Arabia, caused by Middle East respiratory syndrome coronavirus (MERS-CoV). Based on phylogenetic analyses, both MERS-CoV and SARS-CoV originated in bats. Bats are thought to be a natural source of coronaviruses [6]. SARS-CoV-2 has been demonstrated to have 96% genomic homology with bat coronavirus RaTG13 and 79.6% genetic resemblance with SARS-CoV [7].

After the rapid global spread of COVID-19 disease, nationally and globally, there has been a considerable rise in mental health issues. Patients and healthcare workers were the first to report the psychological effects of the pandemic, mainly those in direct contact with the affected persons. Due to strict infection control, social distancing, nationwide lockdowns, quarantine, etc., psychosocial distress, and negative emotions have also been reported to occur in the general population. Due to COVID-19 disease, various mental health problems like anxiety, depression, sleep problems, stress, post-traumatic stress, and various other psychological problems are of main concern.

Fear of infection, the impression of danger, financial issues, and the social cut-off were some of the main reasons for mental distress among the general population. Indeed, economic shutdowns have wreaked havoc on economies around the world, particularly in nations where domestic outbreaks are more severe, health services are less prepared, and economic vulnerability is high.

Some of the COVID-19 survivors have been experiencing major post-traumatic stress disorders with varied duration and severity of symptoms. The mood disturbances and depressive symptoms have been found to be more common [8]. The pandemic, however, had a wide-ranging impact on mental health globally, and many people have experienced increased levels of stress, anxiety and cognitive impairments as the primary symptoms of the post-acute COVID-19 syndrome [9]. The cognitive issues thus observed, ranged from difficulties with memory and attention to more severe problems with executive functions like decision-making. The direct effect of the virus on the central nervous system and the psychological stressors associated with the pandemic might be the potential factors that contributed to the mental health and cognitive problems in the COVID-19 survivors.

Considering the psychological consequences of the COVID-pandemic spread, we attempted to conduct a narrative review on the impact of COVID on the mental health of different human population groups stratified concerning age, sex, and profession groups.

We used web resources to collect various articles on depression and Covid to assess its impact on people of different age groups and professions. Searches were made on Pubmed, google scholar, Scopus, and Embase databases by using different keywords like “Depression And COVID”, “Depression And COVID in females”, Depression And COVID in adolescents, “Depression And COVID in students”, “SARS-COV2”, “Mental illness And COVID”, etc. No time limit was set for the search of articles from the various databases. All the articles were assessed manually to have comprehensive knowledge about the essence thereof. All the articles were segregated by age, sex, and professional groups, and a narrative review was performed concerning each group as follows.

### Depression in the general population due to COVID

The COVID-19 disease may have varying effects on the general population's mental health, depending on national health and government policies, as well as public resilience and societal norms [10]. Amid the COVID-19 epidemic, the global prevalence of mental health-related issues among the general population is 28% for depression, 26.9% for anxiety, 24.1% for post-traumatic stress, 36.5% for stress, 50% for psychological distress, and 27.6% for sleep problems [10].

Globally, various online cross-sectional studies were conducted in countries like China (*n* = 1171), Saudi Arab (*n* = 3032), Bangladesh (*n* = 13,654), Malaysia (*n* = 528), Iran (*n* = 8591), Austria (*n* = 560), Japan (*n* = 2,708), Egypt (*n* = 283), Canada (106) and Brazil (*n* = 482) during the COVID-19 pandemic to access the percentage of depression among the general population. Based on the outcome of the studies, the percentage of depression reported is as follows: 22.6%, 20.9%, 43.5%, 28.2%, 15.1%, 31%, 18.35%, 27.9%, 18%, and 70.3%, respectively [11–20].

A study was conducted in the German general population (*n* = 2503) to access loneliness and mental health during the COVID-19 pandemic and it was compared with the same type of study conducted in pre-pandemic time. Anxiety and depression symptoms were seen to have increased from an average of 0.77 (SD = 1.17) and 0.89 (SD = 1.21) in 2018 to 1.05 (SD = 1.31) and 1.14 (SD = 1.23) in 2020. Loneliness, depression, and anxiety were seen to have more effects on women and younger participants [21].

Data from a German (*n* = 1527) and Norwegian (*n* = 1225) population were analyzed with the help of a cross-sectional study. It was found that there is a strong relationship between general mental distress and COVID-19 stress (*r* = 0.55 and *r *= 0.61) for the German-speaking sample and the Norwegian population, respectively). Locus of Control (LoC) had significant moderating effects in both datasets. It was observed that people influencing their own lives did bear the COVID-19 pandemic in a much better way. An external locus of control, on the other hand, is linked to sadness and anxiety symptoms [22].

Similarly, to access the impact of the COVID-19 pandemic on the mental health of the general population, a comparison was made between the populations of Germany and the UK. 25% of the participants from both countries reported psychological symptoms while as 20–25% of the UK and German participants were seen to have symptoms of depression and anxiety [23].

A similar population-based study was conducted in Hong Kong to access the depression, civil unrest, anxiety, and COVID-19 stressors during the acute phase of the COVID-19 pandemic. Unrest-related stress and COVID-19 were linked to a higher incidence of possible anxiety and depression; people who experienced both stressors had a higher prevalence. This pattern remained true whether the person had little or sufficient assets, although the risk of mental illness was significantly higher among those with lower assets [24].

A web-based international study comprised the general population from 13 countries on four continents was conducted during the first wave of the COVID-19 pandemic from May to August 2020. Out of the total 22,330 participants, 36.7% had symptoms of insomnia while the percentage of depression and anxiety symptoms were 23.1% and 25.6%, respectively [25]. 7381 participants from ten different regions of Cameroon participated in an online study to access the fear of COVID-19 and depression during the pandemic. After analysis of the data, it was found that the fear of COVID-19 was 57.4% and only 8.4% of the participants were depressed. As compared with other countries, depression was seen to be less prevalent in the Cameroon population [26].

The impact of mental and psychological symptoms among the population in quarantine for 2 weeks during the COVID-19 pandemic was documented following a case-controlled study design conducted at the department of psychiatry of Shenzhen Longgang Center for Chronic Disease Control in Shenzhen, China mainland in June 2020 encompassing 1674 participants (aged 18 to 65 years) in quarantine for 2 weeks and 1743 age–sex matched controls living in Shenzhen to assess depressive, anxiety, and insomnia symptoms. Population in quarantine showed significantly higher risks of depression (OR: 4.55, 95% CI 3.82–5.41), anxiety (OR: 2.92, 95% CI 2.43–3.51), and insomnia (OR: 2.40, 95% CI 2.02–2.89) when compared to the general population [27].

To show the comparison of attitude, mental health disorders, knowledge, and socioeconomic burden between healthcare workers and the general population, a semi-structured online questionnaire-based study was conducted in Egypt during the COVID-19 pandemic. Non-healthcare workers (non-HCWs) had a lower frequency of obsessive–compulsive disorder (OCD) (28%) and anxiety (30%) than healthcare workers (HCWs) (29% and 32%, respectively). Non-HCWs had higher depression (69%) than HCWs (66.4%). Urban residence, smoking, history of medical illness, young age, female gender, students and low socioeconomic class were significant associated factors [28]. A case-controlled study was conducted to access insomnia, depression, and anxiety symptoms between the general population and the population in quarantine during the COVID-19 pandemic in China. Higher levels of insomnia, depression, and anxiety symptoms were seen in the population in quarantine [28]. Loneliness, history of mental illness, female gender, younger age, students, people in quarantine, smoking, low socioeconomic class, urban residence, health care workers, etc., have been linked with higher levels of insomnia, stress, depression, and anxiety [16, 29–31]. Focusing on gratitude, eating, being older, regular exercising, sleeping, educational status, awareness of the disease, being married, etc., are some of the important factors to prevent depression and various other mental health issues [11, 15, 16, 32].

### Depression in adolescents due to COVID-19

Covid-19 disease manifestation, its prognosis, and its spreading mechanisms were poorly understood during the initial days of its outbreak. As a result, the general public had to face the consequent disease-associated psychiatric outcomes in the form of anxiety and depression. Since its outbreak in China, a series of studies have been carried out to assess the mental health of people, especially adolescents. The first cross-sectional study was conducted to understand the impact of COVID-19 on the depressive symptoms the adolescents within one month after the start of the COVID-19 outbreak in China. This study revealed that high CRIES (Children Revised Impact of Event Scale) scores were associated with major depressive disorder and avoidance of traumatic memories associated with COVID-19 or experience of flashbacks [33]. These workers proposed long-term monitoring of the adolescents to study the impact of COVID-19 on adolescents with major depressive disorders in China. In Taizhou, China, depressive symptoms are more prevalent among adolescents with a poor parent–child relationship as revealed by a study on data from 6435 middle and high school participants [34]. The presence of depressive symptoms and anxiety in children has been further evident from more studies in Chinese children. Data on 1825 Chinese adolescents have revealed the occurrence of psychotic-like experiences (PLEs) in adolescents [35] and interventions are also suggested for the mental health of adolescents. Depressive symptoms due to COVID are found to be more prevalent in the low fearful girls who show high neural reactivity to social reward while the shy/fearful girls who are less likely to engage socially show less depressive symptoms [36]. More depressive symptoms have been observed in female adolescents before and during the lockdown period which is likely to be the result of diminished connection or support or the decreased activity in the lockdown including a decrease in exercise and resulting weight gain, which can contribute to the feelings of depression [37]. There is a high prevalence of depression among children of rural China especially in left behind adolescents than the non-left behind adolescents [38] and show elevated levels of anxiety and depression due to coronavirus fears, negative effect, intolerance of uncertainty, acceptance/tolerance, rumination and suppression [39]. Depressive symptoms have increased among healthy adolescents during the COVID-19 pandemic, while adolescents with early life stress have high but stable depression symptoms with time [40]. Spain has also reported higher levels of depression among children during the COVID-19 confinement although with lower effect sizes [39]. A higher association of anxiety in adolescents with a history of COVID-19 infection was also found in a study involving the adolescents of Kashmir valley [42].

Character strength has been a powerful factor in controlling depression as it is a protective factor that can buffer the effect of stress and it has a negative correlation with depression symptoms due to COVID-19 as revealed by a study on 617 adolescents [43]. Parents’ involvement has also been found to be a protective factor and lower levels of parent–child communication have been found associated with higher levels of depression among adolescents [44].

### Depression in health workers due to COVID-19

There is a considerable effect of the COVID-19 pandemic on the mental health of healthcare workers because of their close contact with infected patients. During the early period of the COVID-19 outbreak, when the infection cases were more in different Chinese provinces, depressive symptoms were reported in 27.65% of the surveyed medical staff [45]. The various reasons for depression in health workers have been analyzed. Insufficient personal protective equipment, prolonged work hours with a heavy workload, fear of infection and spreading it among family members, poor self-confidence, poor occupational safety, reduction in the time devoted to meditation, infrequent physical exercising, social stigma, and rejection are some of the significant factors responsible. However, working in isolation hospitals was not found to be contributing factor to others [46–50]. Various studies revealed the rate of depression prevalence in different countries and the symptoms were seen from mild to severe. In Italy, 62%, Australia 57.3%, Sri Lanka 53.3%, Iran 44.8%, China 39.7%, Bangladesh 39.5%, Tehran 36%, Indonesia 22.8%, Korea 15.1%, the studied respondents with experience of frontline work at COVID-19 showed depressive symptoms [49–56]. Depression was more seen in infected healthcare workers in comparison to non-infected during the pandemic [57]. A positive correlation was found between poor sleep quality or insomnia, burnout, and depression. [52, 58]. The relatively serious psychological problems in health care workers were seen especially in the 20–30 years age group, women, and nurses with low educational backgrounds and low professional titles during the pandemic [51, 59]. According to a comparative study done by Gundogmus and associates (2021) in Turkey to study the comparison of levels of depression between the first and second COVID-19 peaks, the depression levels were found to be increased in the second peak. Among the medical staff, the severity of symptoms was found in nurses, especially the widowed and poor health, with disrupted social life owing to the stigma of exposure to COVID-19, lower optimism of psychology, no constant visiting friends, and those receiving more than 50% of negative and false information every day [45, 60]. 8.7% of the nurses in hospitals in Indonesia showed a prevalence of depression during the pandemic, while the prevalence rate was significantly high up to 10.5% among those facing financial hardship during the pandemic [48]. Some healthcare professionals even had regrets about their profession because of the pandemic and the associated experiences. It was suggested that the satisfaction of money compensation, promotion of healthy behavior like the use of personal protective equipment, provision of sufficient information on the disease, psychological support along with appropriate psychological interventions in the field are the coping factors for better mental health of health workers.

### Depression in adults due to COVID-19

The social distancing measure adopted during the COVID-19 pandemic has prevented the spread of the virus but at the same time, home confinement has resulted in considerable mental health concerns in people. The adult group of the population is one of the most effective groups. An increase in the rate of severe depressive symptoms from 6.1 to 8.2% in young adults was found from the pre-pandemic to the pandemic period [61]. Even adults with depressive symptoms before the pandemic have shown worse mental health. According to the studies, two-in-five studied persons showed depressive symptoms during the pandemic and the causes which were found to be associated with the depression were poor socioeconomic status, low family income, low education level, loss of employment, dependency on the family for living, chronic comorbidity and even more exposure to the COVID-related news are some of the significant factors [62–65]. Older adults also show depressive symptoms. The older adults living in long-term day care centers show moderate-to-severe depression while the older adults attributed who are caregivers have shown increased and persistent depressive symptoms during the pandemic mainly to financial issues [66, 67]. Multi-country studies were also conducted to analyze the mental health of adults in general. According to a study on young adults in Egypt, Ghana, India, Pakistan, and the Philippines significant variations were seen in mental health. The highest depression was found in the Philippines, followed by Egypt, Pakistan, and India, while the least was found in Adults of Ghana. The prevalence of mental depressive symptoms in the adults of Ghana was found to be 12.3% during the COVID-19 pandemic [62, 68]. It is clear from various studies that there is a potential negative impact on the mental health of individuals due to the disruption of psychological routines. With the passage of time and the implementation of broad social policies related to epidemic control, a ray of hope is seen when in a study it was found that the rate of individuals showing depressive symptoms during the initial period of COVID-19 is 30% which has decreased to 20% over the period of four months [69]. Specific interventions toward the working status of the family carers are recommended [70].

### Paternal depression during COVID-19

The prevalence and burden of depression symptoms were also analyzed among parents during the COVID-19 pandemic. The level was high among parents having infants between 0 and 6 months old, children with abnormalities like autism spectrum disorder, children with psychological symptoms, pregnant women and their spouses as well as in postpartum women [71–75]. The rate of paternal depression in mothers was 14.5%, in fathers 6.4% and the relation to partner’s delivery was found to be 13.82% during the COVID-19 pandemic [74, 76]. Pregnancy is a significant transition period in the life of a woman as it is associated with many psychological and immunological changes. Psychiatric morbidities during pregnancy can adversely affect the health of the fetus. Studies have shown that the pandemic has significantly increased the rate of mild and moderate-to-severe depression from the pre-pandemic to the pandemic period among pregnant mothers from 19.94% and 0.55% to 25.8% and 10.36%, respectively [77]. Further, depressive symptoms also vary among pregnant women in various age groups. Women below 30 years of age have reported a burden of depression than their older counterparts as with advanced age, the resilience power increases [78]. The depression symptoms were not seen among the women only during pregnancy, but the rate of postpartum depression has been reported to be 34% among women [73]. Various reasons are associated with paternal depression-like unemployment, poor family functions, and average socioeconomic status. In postpartum women, past neurosis, history of anxiety disorders, inadequate level of assistance from healthcare professionals, lactation problems, and postpartum pain are the reasons for depressive symptoms [79]. While in pregnant women, the association of increased depression rate during the pandemic has been related to unemployment, poor self-rated health, comorbidities and lack of insurance, emotional stress, partial social support, poor education, low income, and duration of marriage [72, 80–84]. Excessive internet use, the spread of unauthentic news during the lockdown policy, and irregularities in exercise routines have reportedly further worsened maternal depressive symptoms [85, 86]. The regulations of the above factors and professional mental health support are necessary to lessen the effect of depression in one of this vulnerable group of the population.

### Depression among students due to COVID-19

Besides causing major physical health concerns, the persisting COVID-19 pandemic has resulted in strict isolation measures and delays in opening schools, colleges, and universities that have indirectly influenced the mental health of students. The difficulties associated with distanced learning, social isolation, financial distress, racial or ethnic discrimination, and concern about COVID-19 infection were found to be the main reasons for depression among students [31, 87]. In addition to these, lifestyle variables, health-related issues, and reduced physical activity due to homestay also showed a positive correlation with depression [88, 89]. Various studies provide data about the significant increase in depression among students studying in different disciplines. In China, the epicenter of COVID-19, depressive symptoms were seen in 26% of the students, 51.82% of the students in Egypt or Germany, and in university students of Bangladesh, Jordan, and Italy the prevalence was found to be 15%, 78.7%, and 72.93%, respectively. 59.8% of the college students closer to graduating showed depression in the US. An increase in depressive symptoms from 21.5 to 31.7% was found in first-year college students in North California [87–94]. Considerable differences in the severity of depression were also seen among students. Among the students of an Italian university, mild depressive symptoms were shown by 19.7%, moderate by 27.1%, and severe depressive symptoms by 23.6% of the respondents. 21.1% of the school students in China showed severity in depressive symptoms, a large proportion of which was represented by senior high school students [95, 96]. Studies also provide evidence that the rate of depression was higher in female students than male students in general while the low social support getting males also have shown a higher level of depression symptoms [93, 96–99]. The prevalence of mental morbidity among medical students has been described for decades, still, significant changes have been found among them during the pandemic [100]. According to studies, 75.2% of the medical students in Egypt and 56.4% of the nursing students in China showed a prevalence of depression [101, 102]. Insomnia and perceived stress were found to be the main reasons for depression in medical students. The presence of symptoms of depression among the students was not only reported to be coexistent with the COVID-19 pandemic but they seemed to be persisting in 55.1% of the students even after one year of the pandemic [99]. The measures which were found protective against depression in students include more social support, contact with family and friends, improvement in sleep quality, and regular physical activity [44, 97, 99].

## Discussion

**Fig. 1:**
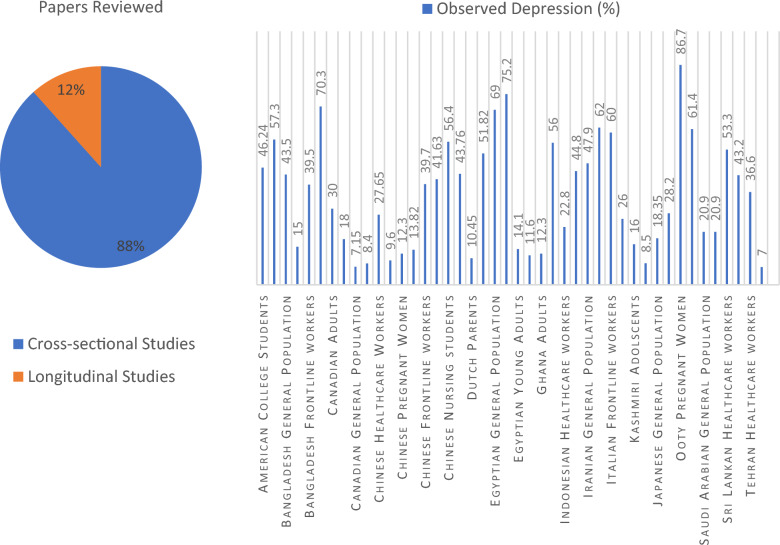
Graphs showing study characteristics

This work is a narrative review of the prevalence of depression following COVID-19 pandemic, its causes, and the ways to manage it among different groups of the population. This study is a narrative review of the global prevalence of depression among various groups of populations. The study has followed the appropriate methods of secondary data analysis for examining nearly 90 related research works. According to our analysis, the prevalence of depression as a result of the pandemic in the general population varies from 6.1 to 70.3% and among the various subgroups of the population varies as well. In adolescents, 16–77.6%; health workers, 27.65–66.4%; adults, 7–61.4%; parents, 6.4–86.7% with the highest among the pregnant mothers; students, 9.6–78.7 having higher rates among medical students. The emergence of COVID-19, its rapid spread have adverse effects on a person’s mental health which can lead to symptoms of depression. Therefore, it is necessary to examine and recognize the mental state of people belonging to different groups of the population during the period. The studies provide us with evidence of the prevalence of depression among people and its severity in different subgroups of the population due to this rapidly transmissible and fatal virus [46, 62, 80, 89]. The studies show different reasons for depression symptoms among different groups of population. In general, low socioeconomic status, history of mental illness, and loneliness are the main factors that show a positive correlation with depression, and female individuals, healthcare workers as well as young age people are more sufferers than others [16, 30, 31]. Students and adolescents face depression symptoms because of difficulties associated with distanced learning, social isolation, reduced physical activity due to homestay, and coronavirus fear (92,95–98). Parents show depressive symptoms due to the reasons like unemployment, poor family function, the spread of unauthentic news during the lockdown policy, comorbidities and lack of insurance, emotional stress, as well as irregularities in exercise routines [72, 80–83]. The frontline health workers showed the most elevated depressive symptoms among all due to insufficient personal protective equipment, prolonged working hours with more workload, fear of infection and spreading it to their families, poor occupational safety, and poor self-confidence [46–50]. After analyzing the severity of depression among the various subgroups of the population, it was found very necessary to adopt strategies to lessen its effect among the people. Various strategies have been suggested by various workers that we help to lessen the effect and can provide insight to work upon so that the challenges like COVID-19 can be dealt with greater efficiency and will not impact the mental health of the individuals. Regulation of the causative factors, providing mental health support, more social support, awareness of the disease, and regular exercise are some of the ways to tackle and lessen depression among individuals in addition to these, the satisfaction of money compensation and promotion of healthy behavior like the use of personal protective equipment’s for the frontline healthcare workers are required. Government and health officials also need to intervene from time to time to refute rumors to reduce the impact of misinformation on the general public’s emotional state and to ensure the adequate supply of personal protective equipment’s and the required infrastructure. These collaborative approaches from both ends will very in tackle the present condition and also provide insights for the future. A summary of the characteristics of included studies is depicted in Table 1 and Fig. 1.

**Table 1:** Summary of characteristics of included studies

S. no.	Author (reference)	Year	Region	Group	Sample size	Depression (%)	Type of study	Mode of study
1.	Abu-Elenin	2021	Egypt	Healthcare workers	237	43.8	Cross-sectional	Online
2.	Adu et al.	2021	Ghana	Adult population	1068	12.3	Cross-sectional	Online
3.	Ahmed et al.	2021	Egypt	General population, Health care workers	524	69, 66.4	Cross-sectional	Online
4.	AlHadi et al.	2021	Saudi Arabia	General population	3032	20.9	Cross-sectional	Online
5.	Ames-Guerrero	2021	Peru	General population	434		Cross-sectional	Online
6.	Antiporta et al.	2021	Peru	Adult residents	57446	61.4	Cross-sectional	Online
7.	Azizi et al.	2021	Iran	Healthcare workers	7626	44.8	Cross-sectional	Online
8. .	Bai et al.	2022	China	Parents	746		Cross-sectional	Online
9.	Baran et al.	2021		Mothers	130		Cross-sectional	Online
10.	Basutkar et al.	2021	Ooty	Pregnant women	60	86.7	Observational study	Direct interaction
11.	Betini et al.	2021	Canada	Adults	3127	> 30	Longitudinal study	Online
12.	Beutel et al.	2021	Germany	General population	2503	> 11.6	Longitudinal study	Face-to-face survey
13.	Chiu et al.	2022	Hong Kong	Older adults	236	56	Cross-sectional	Online, hardcopy questionnaire, Telephonic interview
14.	Colak et al.	2021	Turkey	Pregnant women	149		Cross-sectional	Direct interaction
15.	Couughenor et al.	2021	U.S	College students	194		Longitudinal study	Electronic newspaper
16.	Czysz et al.	2021		Adults	308		Longitudinal study	REDCap electronic data capture system
17.	Fang et al.	2021	China	Healthcare workers	511		Cross-sectional	Direct interaction
18.	Fodjo et al.	2021	Cameroon	General population	7381	8.4	Cross-sectional	Online
19.	Fruehwirth et al.	2021	University in North Carolina	First year college students	419	31.7	Longitudinal study	
20.	Fu et al.	2021	China	Medical staff	7413	27.65	Cross-sectional	Online
21.	Fukase et al.	2021	Japan	General population	2708	18.35	Cross-sectional	Online
22.	Ghio et al	2021	Italy	Health workers	731	62	Cross-sectional	Online
23.	Gildner et al.	2020	United States	Pregnant women	1856		Cross-sectional	Online
24.	Giusti et al.	2021	Italian University	Students	203	Upto 23.6	Cross-sectional	Online
25.	Grumi et al.	2021	Northan Italy	Pregnant mothers	281	26	Cross-sectional	Online
26.	Gundogmus et al.	2021	Turkey	Healthcare workers	2460		Cross-sectional	Online
27.	Guo et al.	2021	Shaanxi province of China	Undergraduate students	1278	Upto 9.6	Cross-sectional	Questionnaire method
28.	Hamaideh et al.	2021	Jordanian university	Students	1380	78.7	Cross-sectional	Online
29.	Herbert et al.	2021	Egypt/Germany	University students	220	51.82	Cross-sectional	Online
30.	Ho-Fung et al.	2022	Sweden	Pregnant women	470	43.2	Cross-sectional	Online
31.	Hollenstein et al.	2021		Mothers and Adolescent children	155 and 146		Longitudinal	Direct interaction and online
32.	Hou et al.	2021	China	Rural Adolescents	826	77.6	Cross-sectional	Direct interaction
33.	Hou et al.	2021	Hong Kong	General population	4011		Cross-sectional	Telephonic interviews
34.	Islam et al.	2020	Bangladesh	University students	476	15	Cross-sectional	Online
35.	Islam et al.	2021	Bangladesh	General population	13654	43.5	Cross-sectional	Online
36.	Jeelani et al.	2022	Indian Kashmir valley	School-going adolescents	426	16	Cross-sectional	Online
37.	Khademian et al.	2021	Iran	General population	1498	47.9	Cross-sectional	Online
38.	Khames et al.	2021		Pregnant women	120		Cross-sectional	Questionnaire method
39.	Khonsari et al.	2021	Iran	Health care workers	938		Cross-sectional	Online
40.	Kim et al.	2021	South Korea	General population	1500	20.9	Cross-sectional	Online
41.	Knolle et al.	2021	Germany and Uk	General population	541, 241		Cross-sectional	Online
42.	Krampe et al.	2021	Norwegian and German-speaking population	General population	1225, 1527		Cross-sectional	Online
43.	Lee et al.	2021	US	College students	200	59.8	Cross-sectional	Online
44.	Lee et al.	2022	First Nations people of Canada	General population	95	18	Cross-sectional	Questionnaire method
45.	Lin et al.	2021	Shenzhen, China	Pregnant women	751	12.3	Cross-sectional	Online
46.	Liu et al	2021	China	Students and their parents	1550		Cross-sectional	Online
47.	Liu and Wang	2021	China	Adolescents	617		Cross-sectional	Online
48.	Liu et al.	2021	China	Medical Students	29663		Cross-sectional	Online
49.	Magnavita et al.	2021	Central Italy	Frontline Workers	153	60	Cross-sectional	Online
50.	Maharlouei et al.	2021	Southwest of Iran	Pregnant mothers	540		Cross-sectional	Online
51.	Marthoenis et al.	2021	Indonesia	Nurses	491	8.5	Cross-sectional	Online
52.	Mistry et al.	2021	Bangladesh	Older adults	1032		Cross-sectional	Telephonic interview
53.	Morin et al.	2021	International- 13 countries and 4 continents	General population	22330	23.1		Online
54.	Noguchi et al.	2021	Japan	Older adults	957		Cross-sectional	Mailed questionnaire
55.	Nomura et al.	2021	Japan	University students	2712		Cross-sectional	Institutional email
56.	Oh et al.	2021	US	College students	36875	Upto 32.68	Cross-sectional	Online
57.	Perera et al.	2021	Sri Lanka	Healthcare professionals	512	53.3	Cross-sectional	Online
58.	Pizarro-Ruiz et al.	2021	Spain	Children and adolescents	590		Cross-sectional	Online
59.	Qi et al.	2021		General population	1171	22.6	Cross-sectional	Online
60.	Rouhbksh et al.	2021	Tehran	Health Care Workers	306	36.6	Cross-sectional	Questionnaire method
61.	Schindler et al.	2021	Germany	Medical students	63		Longitudinal	Questionnaire method
62.	Schmits et al.	2021		Students in Higher education	23307	55.1	Cross-sectional	Online
63.	Shahriarirad et al.	2021	Iran	General population	8591		Cross-sectional	Online
64.	Shehata et al.	2021	Egypt	Young adults	283	Upto 14.1	Cross-sectional	Online
65.	Simon et al.	2021	Austrian	Adults	560		Cross-sectional	Online
66.	Smallwood et al.	2021	Australia	Frontline workers	7846	57.3	Cross-sectional	Online
67.	Soltan et al.	2021	Egypt	Medical students	282	75.2	Cross-sectional	Online
68.	Srifuengfung et al.	2021	Thailand	Older adults	200	7	Cross-sectional	Direct interaction
69.	Sun et al.	2021	Wuhan, China	Parents	1187	13.82	Cross-sectional	Questionnaire method
70.	Sunjaya et al.	2021	Indonesia	Health care personnel	544	22.8	Cross-sectional	Online
71.	Tasnim et al.	2021	Bangladesh	Frontline healthcare workers	803	39.5	Cross-sectional	Online
72.	Van den Heuvel et al.	2022	Dutch	Parents	681	6.4–14.5	Cross-sectional	Online
73.	Villani et al.	2021	Italian university	Students	501	72.93	Cross-sectional	Online
74.	Wang et al.	2021	Chain and Spain	General population	1528		Cross-sectional	Online
75.	Wang et al.	2021	Shenzhen, China	General population	1674		Case-controlled study	Online
76.	Wang et al.	2021		Frontline nurses	498	50.90	Cross-sectional	Online
77.	Wang et al.	2021	Zhejiang Province, China	Adolescents	6435	17.7	Cross-sectional	Online
78.	Wang et al.	2021	Three provinces (Heilongjiang, Henan, and Fujian) of China	Parents of children with autism spectrum disorder and normal	1764 & 4962	21.7–31	Cross-sectional	Online
79.	Watkins-Martin et al.	2021	Canada	General population	1039	6.1–8.2	Longitudinal	Questionnaire method
80.	Wu et al.	2021	China	Young adolescents	1825		Longitudinal	
81.	Yang et al.	2021	China	Epidemic prevention workers	1136	39.7	Cross-sectional	Online
82.	Yee et al.	2021	Malaysia	General population	528	28.2	Cross-sectional	Online
83.	Yigitoglu et al.	2021	Turkey	Healthcare staff	435		Cross-sectional	Direct interaction
84.	Zhang et al.	2021	China	Adolescents	90, 107	36	Cross-sectional	Direct interaction
85.	Zhang et al.	2021	Brazil	General population	482	70.3	Cross-sectional	Online
86.	Zhou et al.	2021	China	Pregnant women	1266	41.63	Cross-sectional	Questionnaire method
87.	Zhu et al.	2021	China	Nursing students	342	56.4	Cross-sectional	Online

Notwithstanding the intervention of governments in mitigating the challenges posed by Covid, populations across the globe have suffered mental health issues in direct or indirect forms. Depression has been reported to be the most serious aftereffect of the pandemic. Be it school and college-going children or working adults, the suffering has been palpable and ominous. In the research paper under reference here, we have tried to highlight the silent menace of depression that accompanied covid pandemic and is still persisting post pandemic. Our effort is to put the qualitative and quantitative proportions regarding the depression caused as a fall-out of COVID in public domain so that this issue is taken up more seriously by all the stakeholders thereby sensitizing the government agencies to encourage counselling. The far-reaching scientific electronic and print media can be the biggest savior with regard to depression in these testing times and this complements the essence of our present research communication well.

## Limitations of the study

Several limitations were noted while reviewing the findings of different studies. Firstly, the study design of most of the conducted studies was cross-sectional and represents the data at a certain time while longitudinal studies help in the better assessment of data over time and can validate the results. Secondly, the small sample size of some of the reviewed studies might limit the generalization of results to a large population. Thirdly, most of the studies used online methods for sampling. And this use of self-reporting instead of diagnostic interviews may lead to biasness. Fourthly, the depressive symptoms among different groups of populations residing in different geographical areas could be influenced by many other variables in addition to the variables considered in the different studies and need to be taken into consideration.

## Conclusion

The present review has indicated that the COVID pandemic has an impact on the mental health of all the population groups irrespective of the sociodemographic variations owing to different countries.

## Data Availability

All the data have been included in the manuscript.

## Abbreviations

CoV: Corona virus
COVID-19: Corona virus disease 2019
CRIES: Children Revised Impact of Event Scale
HCoV: Human Coronavirus
HCW: Health care workers
ICTV: International Committee on Taxonomy of Viruses
MERS: Middle East respiratory syndrome
nCoV: Novel Coronavirus
PLEs: Psychotic-like experiences
SARS: Severe acute respiratory syndrome
WHO: World Health Organization
